# Rapture facilitates inexpensive and high-throughput parent-based tagging in salmonids

**DOI:** 10.1371/journal.pone.0239221

**Published:** 2020-11-11

**Authors:** Michelle Y. Pepping, Sean M. O’Rourke, Connie Huang, Jacob V. E. Katz, Carson Jeffres, Michael R. Miller

**Affiliations:** 1 Department of Animal Science, University of California, Davis, California, United States of America; 2 Center for Watershed Sciences, University of California, Davis, California, United States of America; 3 California Trout, San Francisco, California, United States of America; National Cheng Kung University, TAIWAN

## Abstract

Accurate methods for tracking individuals are crucial to the success of fisheries and aquaculture management. Management of migratory salmonid populations, which are important for the health of many economies, ecosystems, and indigenous cultures, is particularly dependent on data gathered from tagged fish. However, the physical tagging methods currently used have many challenges including cost, variable marker retention, and information limited to tagged individuals. Genetic tracking methods combat many of the problems associated with physical tags, but have their own challenges including high cost, potentially difficult marker design, and incompatibility of markers across species. Here we show the feasibility of a new genotyping method for parent-based tagging (PBT), where individuals are tracked through the inherent genetic relationships with their parents. We found that Rapture sequencing, a combination of restriction-site associated DNA and capture sequencing, provides sufficient data for parentage assignment. Additionally, the same capture bait set, which targets specific restriction-site associated DNA loci, can be used for both Rainbow Trout *Oncorhynchus mykiss* and Chinook Salmon *Oncorhynchus tshawytscha*. We input 248 single nucleotide polymorphisms from 1,121 samples to parentage assignment software and compared parent-offspring relationships of the spawning pairs recorded in a hatchery. Interestingly, our results suggest sperm contamination during hatchery spawning occurred in the production of 14% of offspring, further confirming the need for genetic tagging in accurately tracking individuals. PBT with Rapture successfully assigned progeny to parents with a 98.86% accuracy with sufficient genetic data. Cost for this pilot study was approximately $3 USD per sample. As costs vary based on the number of markers used and individuals sequenced, we expect that when implemented at a large-scale, per sample costs could be further decreased. We conclude that Rapture PBT provides a cost-effective and accurate alternative to the physical coded wire tags, and other genetic-based methods.

## Introduction

Pacific salmon provide one of the most important fisheries in the North Pacific. Fisheries managers from countries around the Pacific Rim must balance ecosystem function with demands from commercial, recreational, and tribal fisheries. In California, Oregon, and Washington, wild-caught salmon generated 200,000 jobs and $12 billion USD in 2012 [1]. Recreational salmon fishing in these states attracted 1.6 million anglers and generated over $1 billion USD [1]. Salmon are also culturally important to many indigenous peoples [2–5], and as a vital food source, salmon are ritually honored with ceremonies and dances [6, 7]. Finally, salmon play a critical role in supporting the ecological function of watersheds throughout the Pacific Rim, where river and forest ecosystems have been shown to be dependent on the annual deposition of marine-derived nutrients from salmon carcasses [8, 9].

Variation within and between salmon species poses unique challenges for management. As anadromous fish, salmon develop in freshwater systems, migrate to the ocean where they complete growth and development, and return to their natal streams to spawn [10]. Both in freshwater and marine habitats, species and stocks of salmon are mixed and can cross state and national boundaries [11]. Most Pacific salmon are semelparous, such as Chinook Salmon *Oncorhynchus tshawytscha*, while others are iteroparous such as steelhead *Oncorhynchus mykiss* (anadromous Rainbow Trout) and require more complex fishing and hatchery regulation [12]. Management groups must communicate and cooperate with each other in addition to other interest groups such as those representing commercial, tribal, and recreational fisheries [13]. Lastly, state and federal regulations, protections, and listings must be followed and are continually changing.

Unfortunately, even as Pacific salmon are increasingly sought after, wild populations continue to experience massive declines [13]. Today, the biomass of returning wild salmon from Washington to California is only 6–7% of the historic quantity [14], and one third of the extant populations are currently listed as threatened or endangered [15]. Declines in wild Pacific salmon have primarily been attributed to overfishing and anthropogenic habitat alterations including dam construction, water withdrawals, logging, farming, and urbanization [16–19]. With the widespread decline of Pacific salmon throughout their range has come a proliferation of fishing and environmental regulations, protections, and conservation status listings. Hatcheries around the Pacific rim artificially spawn returning adults and raise juveniles prior to release into rivers in an attempt to supplement wild Pacific salmon stocks and mitigate for loss of habitat [16]. In 2017, hatcheries in the United States and Canada released over three billion salmon smolts [20].

In order to comply with with regulations and manage stocks, many groups track wild and hatchery fish. Many hatcheries in California, Oregon, Washington, and Idaho mark their fish with a permanently clipped adipose fin to differentiate hatchery-reared and wild-origin fish for informing management decisions, stock assessments, and research [21]. Additionally, many Pacific salmon are marked with external or implanted physical tags. The most common physical tagging method, the coded-wire tag (CWT), is implanted in 50 million hatchery juvenile Pacific salmon annually [22], and when recovered from adult fish, allows the hatchery of origin, date and time of release, and other meta data to be retrieved [21]. Drawbacks of CWTs include high costs, variable implant success, low retention rates, stress induction with placement, the need for killing individual fish in order to retrieve the tag, and a potential lack of statistical power for accurately estimating key exploitation and escapement rates [23]. Another type of physical tag, the passive integrated transponder (PIT), uses radio frequency identification to track individual fish and their movement through permanent antenna installations without handling fish. However, they require specific physical conditions and intensive maintenance of antennae arrays to be effective, so they are not widely employed for population estimates of anadromous stocks [24]. Fish must be raised to a minimize size relative to the tag (e.g., CWT or PIT) that will be implanted before they can be safely tagged [25]. Not only does this potentially create sampling bias for larger fish, but it also can be expensive when accounting for food and other hatchery costs. Implementing new technologies could improve the cost and efficiency of information that is currently being collected by physical tagging [13].

Using genetic information to track individual fish is rapidly becoming a powerful alternative to physical tagging. Genetic tracking methods have the potential to increase the number of individuals marked, enhance the amount and type of information collected from each individual, minimize stress on tagged fish, and decrease cost of current physical tagging methods. In addition, genetic tracking methods allow for life-long marker retention, no minimum size for tagging, nonlethal detection, and high resolution differentiation of individuals. In addition to tracking individuals, genetic methods can also yield relevant data on the relatives of sequenced individuals [26–29], thus allowing the potential assessment of population structure [29–32]. A small number of independently segregating microsatellite markers have been successfully used in pedigree reconstruction, but are constrained by inflexibility between species and difficulty designing markers [32, 33]. Single nucleotide polymorphisms (SNP) are being increasingly used as genetic tags because they are high-throughput, have low genotyping error, and their cost of detection is continuously decreasing [29, 30, 34, 35]. Previous challenges associated with SNPs in forensic analysis, such as needing high concentrations of DNA for sequencing, are no longer relevant [36]. SNP-based genotyping methods include whole genome sequencing, reduced representation sequencing such as restriction-site associated DNA (RAD) sequencing, Amplicon sequencing (e.g., Genotyping-in-Thousands), SNP chips, and PCR assays (e.g., SNP Type or Taqman) [34, 37–42]. Parent-based tagging (PBT) is a method of genetic tracking that can use any of these technologies to identify the offspring of specific individuals (e.g., hatchery broodstock) from genetic information [29, 30, 32–35].

Genetic tracking differs from physical tags in its limitations, type of information collected, and amount of information. Genetic tracking is limited by the technology available such as marker design as well as applicability across species and management groups [34, 36, 43, 44]. Genetic tracking using SNPs discovered and genotyped via sequencing of restriction-site associated DNA tags (a.k.a. RAD sequencing or RADseq) has the potential to offer increased flexibility when compared to SNP chips or Amplicon sequencing. Though there are drawbacks of interrogating thousands of RAD tags per individual such as, high expenses and increased data complexity, a large panel of SNPs is often unnecessary and studies have used less than 100 SNPs for accurate parentage assignment of the vast majority of individuals [30, 35].

A molecular method which targets specific RAD tags in the genome may offer accurate and inexpensive PBT and other genetic tracking approaches. Here we test a new method of genetic tracking, Rapture-PBT, that accurately and efficiently constructs pedigrees from Rapture SNP data. Rapture [42] is a combination of RAD and capture sequencing which facilitates the inexpensive genotyping of large numbers of individuals across hundreds to thousands of loci. Rapture achieves high sample multiplexing at low sequencing cost by using RNA baits for targeted enrichment through hybridization-based capture of a subset of RAD tags prior to sequencing. We tested Rapture-PBT on 572 adult fall and late fall Chinook Salmon and 730 progeny from a subset of 232 fall run adults (116 known spawning pairs) from the Coleman National Fish Hatchery, Battle Creek, California. Fish released from this hatchery are intended to assist in mitigating for the ongoing loss of Central Valley salmon habitat. We found that Rapture-PBT successfully assigned the progeny to parents with a 98.85% accuracy and a cost of approximately $3 USD per sample with 248 SNPs from 500 baits.

## Results

We wanted to test the application of Rapture-PBT as well as the utility of the baits across different species within the *Oncorhynchus* genus. Starting with 500 baits previously designed for Rainbow Trout [42], we decided to test Rapture-PBT on Central Valley Chinook Salmon. Chinook Salmon and Rainbow Trout are as divergent as any species pair within the *Oncorhynchus* genus [45]. Additionally, Central Valley Chinook Salmon are currently an important stock of salmon that could benefit from the implementation of a genetic tagging method. Adult fin clip samples were taken, and embryo tissue was dissected from eggs. We then extracted DNA, generated RAD libraries, performed capture on the libraries using Rainbow Trout baits, and sequenced the resulting Rapture library [42, 46] (see Methods). This dataset should allow for a thorough evaluation of Rapture-PBT using a cross-species bait set.

**Fig 1:**
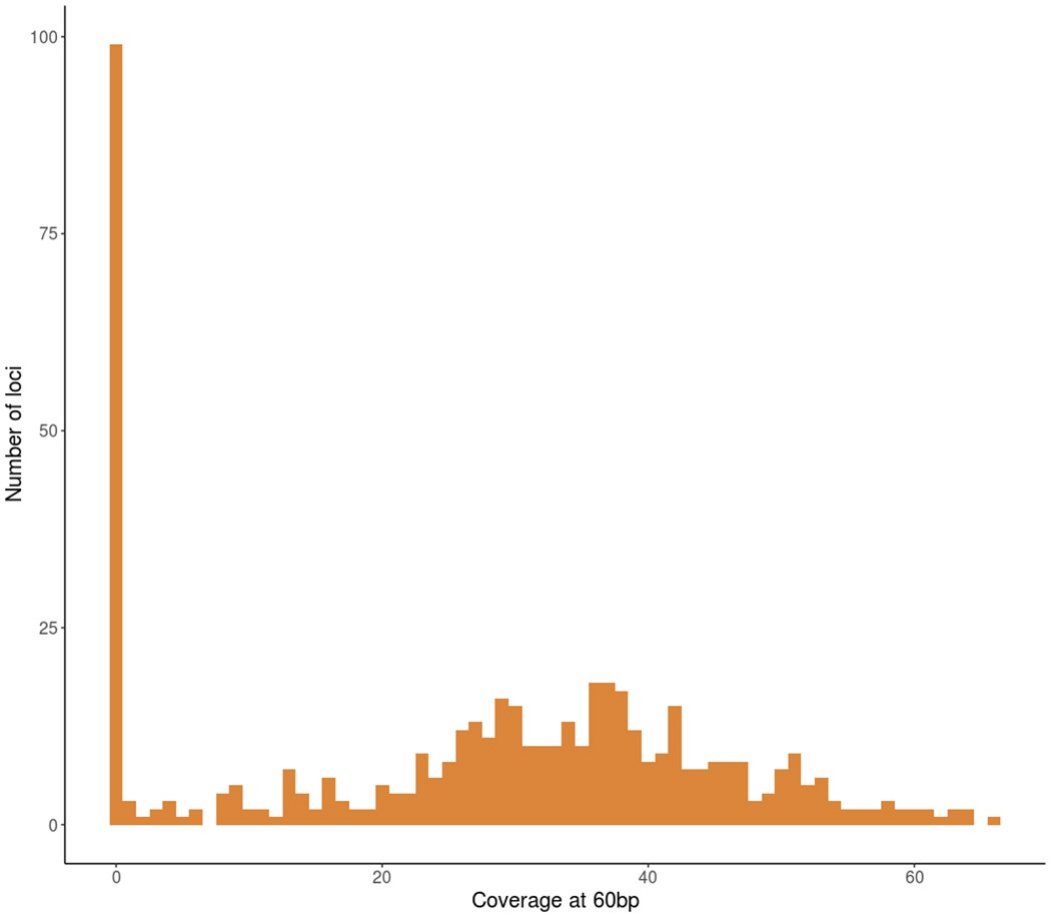
Rapture loci sequencing coverage. Histogram of the mean coverage of all 500 bait-targeted loci at the midway position. The midway position was 60bp in the 5’-3’ direction from the start position of the capture bait.

**Fig 2:**
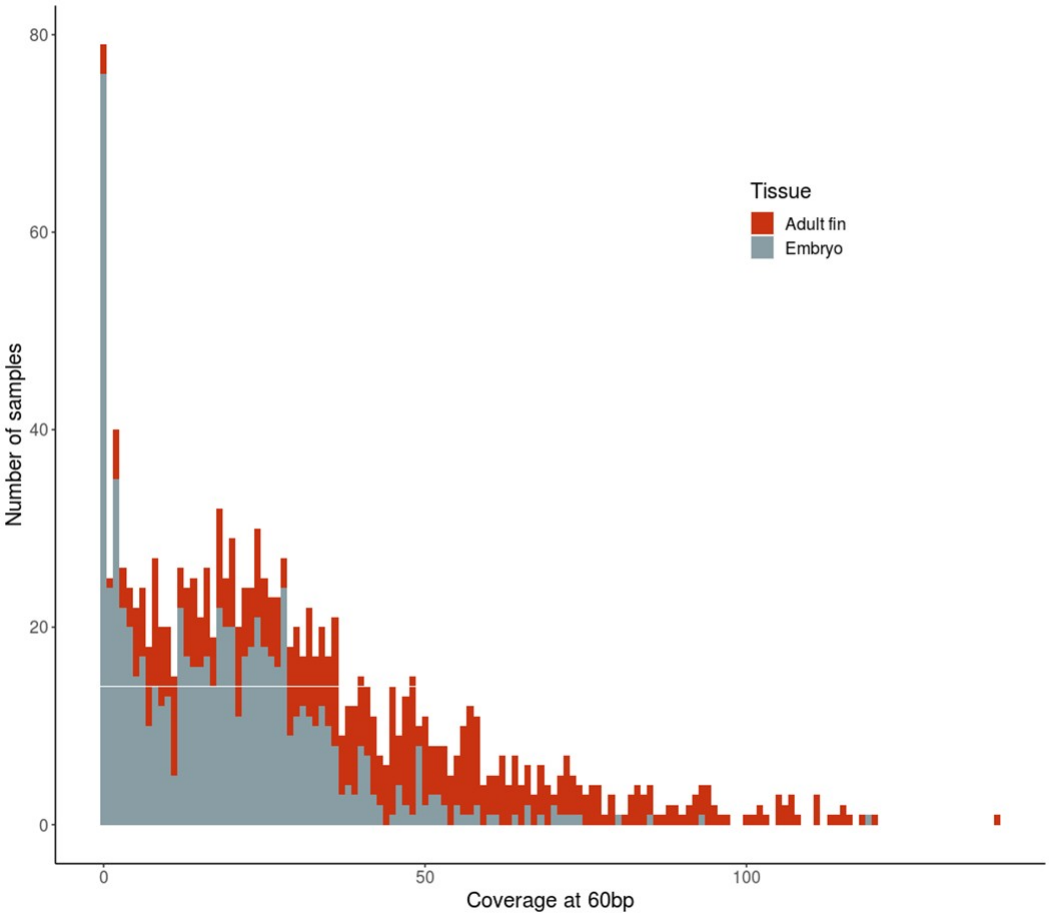
Sample sequencing coverage. Histogram of the mean coverage of samples at midway position of all 500 bait-targeted loci. The midway position was 60bp in the 5’-3’ direction from the start position of the capture bait.

To begin evaluating our Rapture-PBT dataset, we examined coverage, or depth, across samples and loci. The 500 bait-targeted loci had a mean coverage of 27.3X across all individuals (Fig 1, S5 Table). However, the distribution was bi-model. Approximately 400 of the 500 targeted loci had good coverage, while approximately 100 Rapture loci failed (i.e., had less than 1X mean coverage). The majority of Rapture loci had high coverage: 394 Rapture loci had a mean coverage over 4X, 380 Rapture loci had over 10X mean coverage. We also evaluated coverage of all 1,302 samples (Fig 2, S6 Table). One hundred samples had sequence coverage less than 1X, and 181 samples had coverage below 4X. Conversely, 1,121 samples had coverage over 4X, and 988 samples had coverage over 10X. We conclude that these baits, even though designed for Rainbow Trout should provide ample loci for parentage assignment in Chinook Salmon. Additionally, the majority of samples should have sufficient data for parentage assignment.

To discover Chinook Salmon SNPs for use in parentage assignment, we used the probabilistic framework implemented in the software package ANGSD on the 394 Rapture loci with greater than 4x coverage [47] (see Methods). We retained 248 final SNPs, each on a separate Rapture locus, that passed filtering requirements for use in parentage assignment (S8 Table). These 248 SNPs identified from Rapture should be sufficient for parent-based tagging in Chinook Salmon.

**Fig 3:**
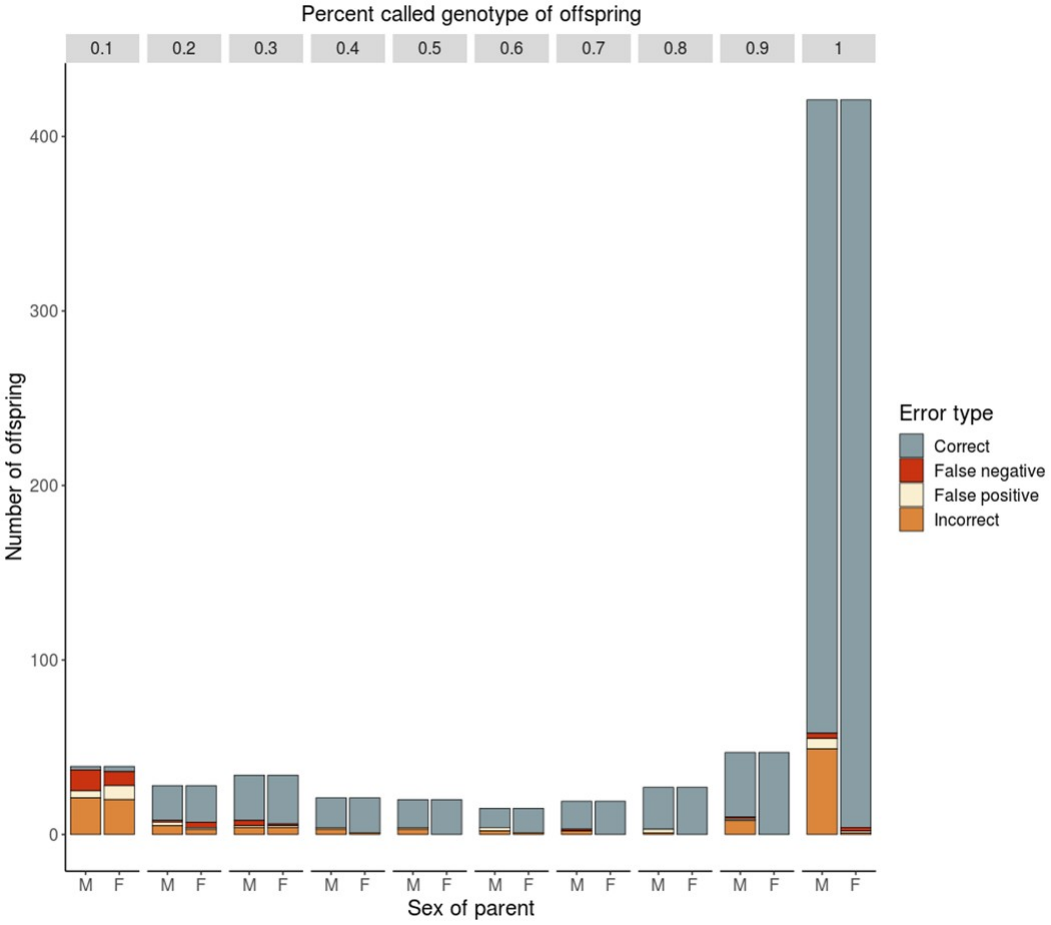
Error analysis of parentage assignments. Stacked bar plot of assignments of parents to offspring compared to the observed hatchery spawn; grouped by closest tenth of a percent of called genotypes of offspring; errors separated by sex of parent M—male, F—female; shade indicates error type: correct, false negative (no parent assigned when one was present), false positive (parent assign when not present), and incorrect (parent assigned to incorrect parent when parent present).

To test the utility of SNPs for parentage assignment, we called genotypes and used the COLONY parentage assignment program [48] to assign family structure. Due to the 1:1 spawning scheme used at Coleman and many other hatcheries, hatchery parentage assignments often assume monogamy. However, we elected to not assume monogamy to allow for all parent-offspring possibilities. We input fall- and late fall-run adults with >80% called genotypes (198 SNPs, 478 adults) and all offspring individuals with >1% called genotypes (two SNPs, 671 offspring), as we also wanted to evaluate the minimum number of called SNPs in offspring necessary for successful parentage assignments (S9 and S10 Tables). Parentage assignment fell into four categories (Fig 3, S13 Table). The majority of offspring with <10% genotypes were assigned incorrectly (Fig 3). Offspring that range 10%-30% called genotypes have mostly correct parentage assignments, but have significant errors, especially false negatives. Offspring with >30% called genotypes (74 SNPs) have consistently low error rates. Interestingly, however, the male parent misassignments were significantly higher (Fig 3). Male bias was even present in offspring with >80% called genotypes, which lead us to suspect a biological basis rather than a technological one. We conclude that SNPs discovered through Rapture worked well for PBT even in offspring with relatively low amounts of data; however, the sex-biased pattern of misassignments warranted further investigation.

**Fig 4:**
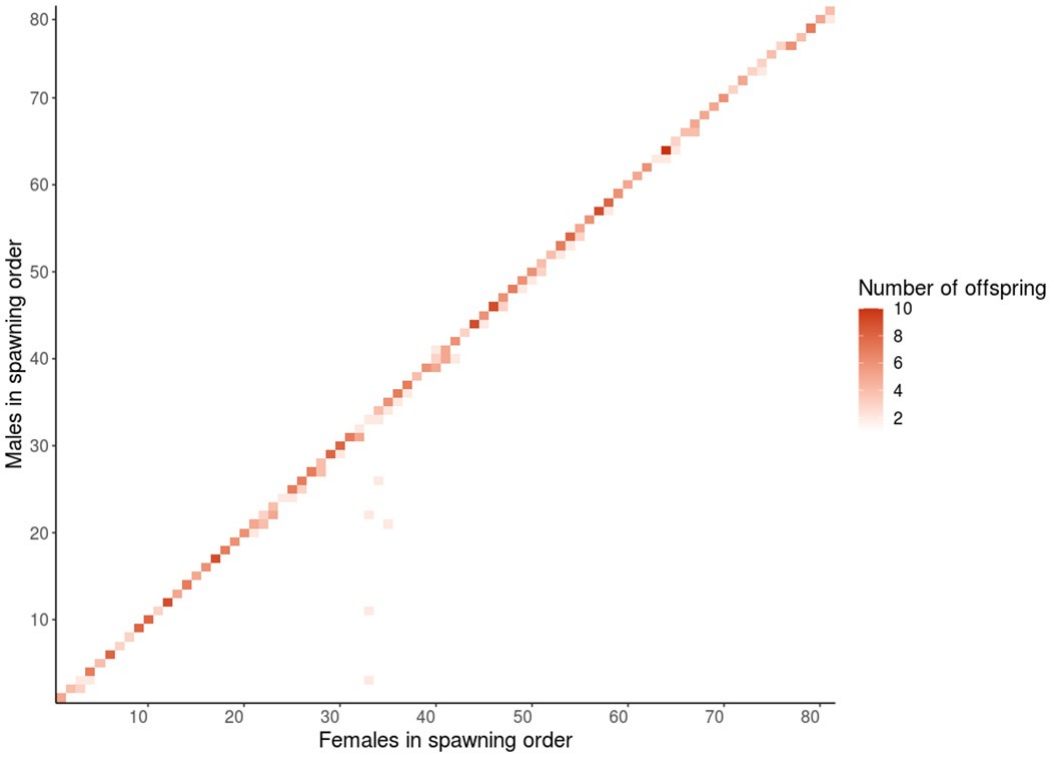
Parent pair output in spawning order. Heat map of the number of offspring for every possible parent pair; shade indicates number of offspring produced by parent pair according to COLONY output.

**Fig 5:**
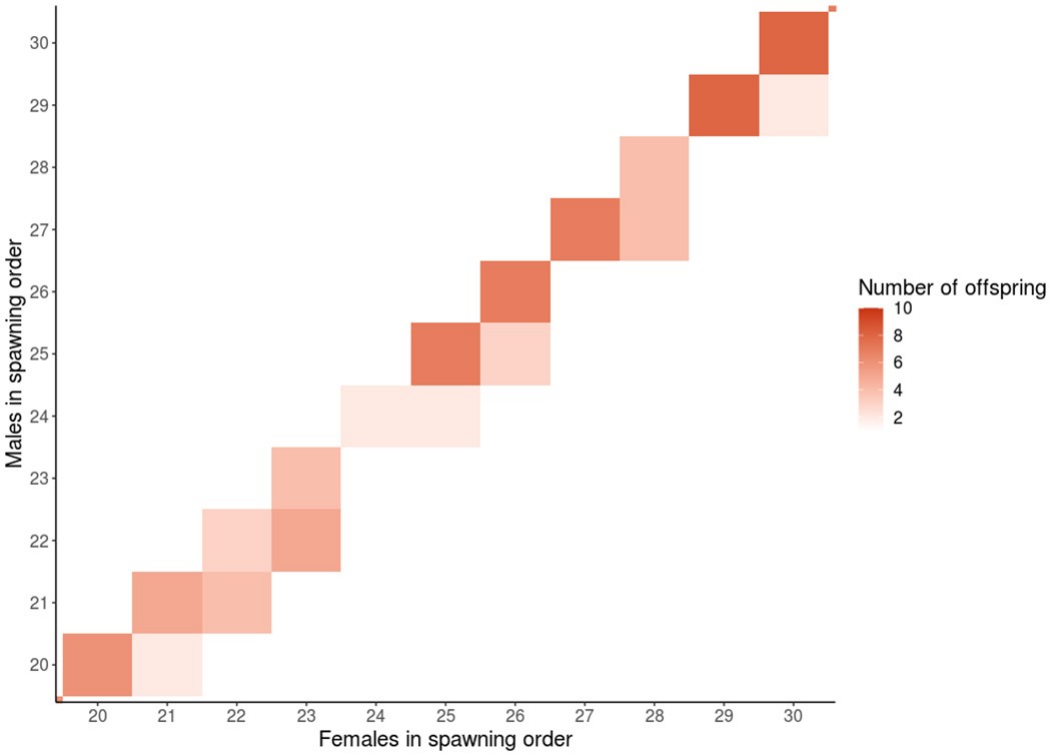
Enlarged parent pair output in spawning order. Heat map of the number of offspring for possible parents pairs from adult 20–30; shade indicates number of offspring produced by parent pair according to COLONY output.

To determine if the offspring were parented by an adult outside of the recorded spawning pairs, we analyzed the assignments by spawn order in the hatchery. There is a difference between male and female errors for individuals above 30% called genotypes (Fig 3), where there was a total of 89 incorrect male assignments and 18 incorrect female assignments, with many misassignments of offspring with ample genotype data. Strikingly, 80 of the incorrect males were assigned to the male of the previous spawning pair, which suggests milt contamination from previously spawned males (S16 Table). In Fig 4, the direct diagonal contains the offspring from the observed spawn, all other offspring were first deemed as incorrect assignments. There is a pattern where almost all assignments off the diagonal fall to the right-hand side, suggesting these errors are likely true assignments due to male contamination from residual milt (Figs 4 and 5). If the assignments are correct, male contamination from the previous spawn occurred in 14% of all recorded spawns (S13 and S16 Tables). Additionally, 12 of the female parent misassignments were tracked down to one event of the switching of two adult samples in neighboring wells during molecular biology, and two were also found to be from the previous female spawned (S16 Table). Considering only the 570 offspring with over 30% called genotypes, parents with over 80% genotypes, and removing the errors caused by male contamination from the previous spawn, male parents were assigned with an accuracy of 98.42% and female parents were assigned with an accuracy of 99.30%. There was a false negative rate of approximately 0.35% for male and 0.18% for female assignments. We conclude that Rapture-PBT was very accurate overall, and this is a conservative accuracy rate considering that gametic contamination was likely more prevalent.

To simulate parentage assignment on returning adults, where it is likely that only a subset of samples would have genotyped parents, we removed parents to increase the number of offspring without parents input into the COLONY program. We ran the program including 671 offspring with at least 2 called genotypes, 10 known parents, and the remaining adults with no offspring. The adults all had a minimum of 80% called genotypes and consisted of 65 late fall-run adults, 222 fall-run adults with no offspring, and 10 fall-run (5 male and 5 female) with offspring. We purposely used a small number of correct parents to simulate a real-life situation where offspring samples would be taken as returning adults 3 years after parental samples were collected. For example, in some reasonable scenarios, very few of the returning individuals would be offspring of tagged broodstock. Additionally, we decided to incorporate late fall-run individuals into the test in order to determine if the program had more difficulty assigning related individuals when members from a different run were present. Of the 570 offspring with greater than 30% called genotypes (74 SNPs), there were only two offspring assigned to incorrect male and 3 offspring assigned to incorrect female parents. In other words, 99.56% of the assignments of parents to offspring were correct. The incorrect assignments were to adults in the fall-run as opposed to late fall-run. Thus, COLONY did not assign any offspring to parents in a different population and had a very low rate of incorrect assignments, even when most potential offspring did not have included parents.

To assess the feasibility using of Rapture-PBT in a large scale, we calculated all of the costs per sample for molecular work and sequencing. The molecular techniques for DNA extraction, RAD library construction, and capture were $2.60 per fish sample (S17 Table). By only interrogating a subset of the RAD tags through Rapture, sequencing costs are lowered to only a small fraction of the total cost. By using 50% of an Illumina HiSeq 4000 lane, sequencing costs were $0.53 per sample, making the total cost $3.13 per sample (Table 1, S17 Table). Hammer and Blankenship conducted an in-depth comparison of cost for various methods of marks, tags and combinations used specifically for monitoring salmonids, such as Chinook Salmon, in 2001. They found that CWTs cost $0.60–0.136 per fish for application with an additional $3–5 per fish to decode the tags, and PIT tags cost $2.50–5.50 for application with negligible decoding costs after equipment associated costs were removed [49]. Similar to that study, our cost analysis does include laboratory materials, but not equipment costs. Rapture-PBT is slightly more cost effective, but similar to Genotyping-in-Thousands, a version of Amplicon sequencing, which costs $3.98 per sample [41].

**Table 1:** Cost of reagents and sequencing for Rapture-PBT. Cost of each molecular step in Rapture-PBT by 96-well plate and individual sample. Cost does not include labor or equipment.

	Cost per 96-well plate	Cost per Sample
DNA Extraction	$84.34	$0.88
RAD Library Preparation	$116.48	$1.21
Capture Step	$48.61	$0.51
Sequencing	$51.00	$0.53
Total Cost	$300.47	$3.13

## Discussion

Our results suggest Rapture-PBT is a powerful, flexible, and efficient method of parent-based tagging. Rapture is flexible through targeting specific RAD tags, where the number and location of sequenced regions can be easily changed. The bait set can be expanded to increase the number of informative SNPs or reduced to decrease cost per individual. Rapture also allows targeting RAD tags from specific genomic regions of interest, such as the *GREB1L* region [46]. By sampling a subset of the genome with Rapture, we obtained sufficient SNPs for parentage assignment. In this experiment, Rapture was not executed under optimal conditions (e.g., we used a bait set designed in Rainbow Trout for Chinook), highlighting the flexible specificity of genome sub-sampling using capture baits. Even with a limited number of baits designed for a related species, Rapture was sufficient for accurate parent-based tagging and yielded more informative SNPs than necessary. We have found 500 baits to be flexible and applicable to a wide range of projects [50, 51]. Other groups have had success with up to 16,000 baits [52]. Given the increases in sequencing capacity, we would recommend going forward designing many baits, at least 1000 baits.

It seems likely that the 100 Rapture loci with low coverage failed for technical reasons because the baits were designed for Rainbow Trout, especially given that the vast majority of these baits produced high coverage in Rainbow Trout [42]. The baits producing low coverage are likely due to polymorphisms in the Chinook Salmon genome that disrupt the restriction site in the targeted loci. Although Chinook Salmon and Rainbow Trout are as divergent as any species pair within the *Oncorhynchus* genus, they were still similar enough to use the same baits with ample data for parentage assignment. Based on the success of this pilot experiment, we propose the possibility of preforming Rapture-PBT on all Pacific salmonids using the same bait set. Not only would these baits be more than sufficient for accurate parentage assignment, but also the sequence data could be informative for future comparisons between species in the Oncorhynchus genus. Although the 500 Rainbow Trout baits would likely be sufficient for pedigree reconstruction for all species in the Oncorhynchus genus, an improved bait set could be designed more specifically from RAD data and multiple species if desired. Thus, Rapture-PBT would be an elegant and straightforward method for large-scale implementation of genetic tracking.

Rapture-PBT not only has the potential to alleviate many of the challenges faced by CWTs, but also is cost-effective. Physical tags like CWTs have drawbacks including poor tag retention, stress induced by tagging, and lethal detection. Though CWTs have already been scaled up to a substantial level, and PIT tags give useful migration data, they both lack information that can be provided by genetic analysis. Additionally, physical tags have costs related to raising fish to a relatively large size before tag implantation [25]. As sequencing costs continue to decrease, the feasibility of large-scale genetic tagging will increase. With a bait set designed for Chinook Salmon, a smaller number of baits could be used to decrease the cost per sample. Utilizing Rapture SNP data for PBT lowers the price so that it is competitive with physical tagging prices. Additionally, tissue would only be collected from adults, which have a significantly smaller sample size than any physical tagging methods that are implemented on juveniles. At $3.13 per samples, Rapture-PBT is competitive with the cost of CWT and PIT tags and yields data on individuals which PIT tags and CWTs are not suited to provide.

After completing parentage assignments, we discovered a novel biological aspect of hatchery practices. Many hatcheries including Coleman National Fish Hatchery implement a 1:1 pairing scheme where the eggs of one female are crossed with the milt from one male. We used this scheme to construct our expected parentage assignments, where the offspring from each spawning event would be from the known crossed parents. Our study revealed that there is more diversity than previously assumed with the 1:1 spawning scheme since many offspring were fathered by residual sperm from previous spawns. We only accepted assignments of individuals fathered by the immediately previous spawn as correct, but given the sex-biased mis-assignment rate and asymmetry in assignment order, it is likely the offspring assigned to fathers from earlier spawns are correct as well. Thus, we suspect that the assignment error rate in males is much lower than our conservative calculation.

Fertilization from many males has implications for past and future pedigree data. Families tracked with external tags are under the assumption that the individuals that were attempted to be crossed are the parents of the tagged juveniles, which could be incorrect in a significant portion of individuals. Because we conservatively assumed that only fertilizations from the single previous male were correct, we suspect true rates of sperm contamination to be higher than the 14% we calculated. Studies that use external tags to track relatives from a hatchery with similar spawning practices should consider the possibility of unexpected gamete mixing. This also reveals that future PBT with hatchery samples should not assume monogamy when inputting data into parentage assignment software. Fertilization is a molecular event with great consequences that can be revealed with molecular techniques. Unpredictable results from manipulated spawning confirms the need for genetic marking and analysis for managed species at the individual level.

To improve Rapture-PBT going forward, we suggest more consistent extractions of DNA, and an improved bait set to increase the number of informative SNPs used for PBT assignments. There was unexpected variation in sequencing success in the offspring we analyzed due to inconsistent DNA extractions among embryo tissue samples. Though only a fraction of the embryo was used for DNA extraction, this tissue produced a high concentration of DNA, which created problems with a magnetic bead DNA purification. Our most consistent embryo DNA extraction was from a plate where the lysate was diluted 50:50. In hindsight, a 50:50 dilution on all embryo lysate could have been used to promote more uniform DNA concentrations after extraction. In practice, Rapture-PBT would utilize DNA extracted from adult fin clips, which do not have the inconsistencies that we experienced with embryo tissue, thus yielding more consistent sequencing coverage across samples. We predict that implementation of Rapture-PBT in a management situation would have more accurate assignments due an increase in the number of called genotypes per individual from fin-clip DNA extractions.

High-throughput genetic analysis with low sequencing costs has far-reaching and immediate implications for fisheries management and conservation. When reproducing adults are genotyped, the information can be used as a unique identifier for individuals who share a significant portion of genes with that individual such as offspring and siblings [34]. Rapture could also be used to discern sibling relationships to estimate the number of spawning individuals in wild populations. The methods used here could be informative for hatchery broodstock and wild population introgression through monitoring of hatchery offspring and grandoffspring potentially spawned in the wild. Studies have shown a rapid decline in reproductive success in the wild after one or few generations of being spawned in a hatchery [53, 54]. Rapture-PBT would be an excellent tool for specifically monitoring reproductive success from hatchery, wild, and mixed stocks.

A case study that could benefit from Rapture-PBT is research of juvenile Chinook Salmon reared on inundated floodplains in the Central Valley of California. Floodplain reared juveniles exhibit increased growth rates compared to individuals confined to adjacent river channels [55, 56]. Gaining a better understanding of how these habitat-specific juvenile growth rates effect eventual rates of the return of adult Chinook is a matter of pressing conservation concern best answered by the release of genetically tracked groups of juvenile salmon into adjacent floodplain and river channel habitats. Only a small fraction of juvenile salmon survive to return as adults. Therefore, each release group must be comprised of hundreds of thousands of tagged juvenile salmon to ensure recapture of sufficient numbers of returning tagged adults. To date, the State of California mandates that all juvenile salmon used for research be implanted with a CWT has made procuring adequate numbers of experimental fish prohibitively difficult [57]. Rapture-PBT can effectively and economically track this same large number of fish through their entire life history by only sampling broodstock and returning adults. Rapture-PBT will also allow us to identify the specific family groups or genetic backgrounds from which successful individuals originate. Taken together, our results demonstrate that Rapture-PBT is an affordable option to track many individuals at low cost and provides a large amount of data per individual.

## Materials and methods

### Sample collection

We collected fin clip samples of fall- and late fall-run Chinook Salmon from Coleman National Fish Hatchery along Battle Creek of the Sacramento River in California. A total of 572 adults were collected, 476 fall-run were collected on October 13, 2016 and 96 late fall-run were collected on January 4, 2016 (S1 Table). All actions done, including clipping of fins and sacrificing embryos with ethanol, were approved under UC Davis IACUC protocol #18883. Fin clips were taken after a male and female pair were spawned together using a funnel that directed eggs and milt into a bucket. Adult fin clips were placed on Whatman paper and dried at room temperature. After each mating, the bucket was replaced and the funnel was rinsed with water. Hatchery personnel spawned fish pairs and incubated the fertilized eggs separately. Approximately half (118) of the fall-run batches of fertilized eggs were marked with a domino that traveled with the eggs through the washing and incubation processes. Eggs were collected on November 3, 2016, after the embryos had developed for 21 days. Individual eggs and batches of eggs had varying quality with some eggs dead. We collected eight embryos from each marked egg batch and transferred them to an individual tube containing 95% ethanol as a fixing agent. Two batches of eggs could not be located, yielding 116 families of parents and 826 eggs (S2 Table).

### DNA extraction

In the laboratory a ~4 mm^2^ piece of adult fin clip was transferred to a well in a 96-well plate for DNA extraction. Embryos were dissected from each egg and individually rinsed in 75% ethanol. We removed the head and transferred it to a 96-well plate for DNA extraction. A total of 730 eggs contained sufficient embryo tissue for DNA extraction. Embryo tissue was placed in 96-well plates containing Lifton’s buffer and frozen at -20°C until all samples were processed. A bead-based protocol was used as described [42] to extract DNA from the 1,302 total tissue samples with an additional heating step of DNA-bound beads in LoTE at 65°C for one minute. Fall-run adult fin-clip DNA was sequenced in triplicate to increase coverage. Embryo DNA had variable concentration and was normalized. DNA was quantified using Quant-iT PicoGreen dsDNA Reagent (Thermo Fisher Scientific) with an FLx800 Fluorescence Reader (BioTek Instruments).

### RAD and Rapture

The first step in applying Rapture is choosing baits. Ali et al. designed 500 baits for Rainbow Trout, that are distributed across the species’ 29 chromosomes (based on their positions in a genetic map) [58]. Rainbow Trout and Chinook Salmon are related species, belonging to the same taxonomic genus and thus have similar genomes, but differ greatly within the *Oncorhynchus* genus [59]. Given that many *SbfI* restriction sites are shared between the two species, in addition to the flexibility of the loci used for genetic assignment, we reasoned that the preexisting baits could also be used for parentage assignment in Chinook Salmon [46]. *SbfI* RAD libraries were prepared with well and plate barcodes using a new RAD protocol [42]. We then used a previously described bait panel of 500 RAD loci (ordered as a MYbaits kit from Arbor Biosciences) for capture [42]. The library was then sequenced with paired-end 150 base pair reads on 50% of an Illumina HiSeq 4000 lane.

### Sequencing analysis and SNP filtering

Rapture sequencing data were demultiplexed by requiring a perfect barcode and partial restriction enzyme site match. We aligned the sequence reads to a recent draft assembly of the Rainbow Trout genome [60] because a suitable Chinook Salmon genome was unavailable [42, 60]. The data for adult triplicate samples were then merged. To account for sequencing errors, we then calculated the of number of times specific nucleotides were sequenced also known as the coverage or depth. We used SAMtools [61] depth function with the bed option to create a file with the depth (number of sequence reads) of each sample at the midway position of each sequenced capture bait-targeted loci. Then, we wrote a simple perl script to calculate the mean for each Rapture locus and each sample. We chose the 60bp location because it is the middle of the 120bp targeted bait (S5 and S6 Tables). We evaluated 4x as a low-medium coverage level and 10x as a medium-high level [62]. We filtered for SNPs and called genotypes using ANGSD version 0.920 [47] with a minimum SNP *P*-value of 10^−12^, a base call accuracy of 99%, and a posterior cutoff of 95%, and a minimum individuals of 50%. We then estimated their minor allele frequency and used a likelihood ratio test to identify two paralogous SNPs (S7 Table). In the 394 RAD loci that greater than 4X mean coverage, we discovered 501 SNPs that passed predefined filtering requirements across 250 RAD loci, and the remaining 144 RAD loci did not contain SNPs that met filtering requirements. Furthermore, after removing two SNPs that appeared to be paralogs, there were 499 SNPs across 248 RAD loci. Lastly, to avoid linkage, we selected one SNP per RAD locus with the highest minor allele frequency. When multiple SNPs were present in one RAD locus, the one with the highest minor allele frequency was chosen (S8 Table).

### Analysis with COLONY program

SNP data was formatted and input into the COLONY program [48]. COLONY version 2.0.6.5 from the Zoological Society of London uses full-pedigree likelihood methods to infer relatedness of individuals using multilocus data with flexible assumptions and data requirements [48]. We assumed no dioecious species, inbreeding, polygamy, no clones, no prior known sibling relationship, unknown population allele frequency, one run, length of run equal to two, full likelihood, and very high precision with likelihood. We then ran COLONY with sequence data on all offspring with at least 1% called genotypes and adults with at least 80% called genotypes (S10 Table). COLONY includes the probability that each assignment is correct All assignments including misassignments were accepted if they had probabilities above 95%. The COLONY input was optimized by running an input file into the program 10 times with 10 different seed numbers and taking the mode of the outputs (S12 Table). We found that two adult samples in neighboring wells had assigned to each other’s offspring. Errors were confirmed when separating triplicate adult sequence data where one plate had inconsistent genotypes with the other two duplicates. These errors were removed before further analysis.

To test a situation where only a small fraction of individuals to be assigned have genotyped parents, this process was repeated. The input consisted of only 10 parents with known offspring, all parents with no known offspring, and all offspring to test a scenario where many individuals do not have genotyped parents. The simulation had the same assumptions as the complete dataset input into COLONY and the output was also run ten times and the mode was analyzed (S14 and S15 Tables).

### Error analyses

We assumed perfect assignment where the adults marked for each spawn were the true parents. There were four levels of error marked for each assigned parent to an offspring: (0) correct assignment, (1) no assignment when parent was present (false negative), (2) assignment when parent was not present (false positive), (3) incorrect assignment with parent present. Errors were categorized by error type, genotype success of offspring and parent, and spawning order (S13, S15 and S16 Tables).

## Supporting information

S1 Table Spawning pairs. Identification and order of male and female adult spawning pairs.
(XLSX)

S2 Table Observed family groups. Observed male and female adult spawning pairs and the eggs collected after the observed spawn.
(XLSX)

S3 Table Sequence counts. The number of sequence reads with multiple filtering requirements for each plated and sequenced well.
(XLSX)

S4 Table Sample identification and merging. Identification of samples sequenced multiple times.
(XLSX)

S5 Table Mean Rapture loci coverage. Coverage of each bait-targeted locus across all samples at the 60bp position of 120bp long reads.
(XLSX)

S6 Table Mean sample coverage. Coverage of each sample across all bait-targeted loci at the 60bp position of 120bp long reads.
(XLSX)

S7 Table All discovered SNPs. All discovered SNPs with information about the position, allele frequency, and number of individuals with sequenced SNP.
(XLSX)

S8 Table Final SNP panel. The location of each SNP used in the final SNP panel.
(XLSX)

S9 Table Number of called genotypes. Number of called genotypes of the 248 SNPs for each sample. Adult samples with less than 198 called and embryos with less than 2 called genotypes were removed for input into COLONY.
(XLSX)

S10 Table COLONY input. Input file submitted into COLONY.
(XLS)

S11 Table Expected COLONY output. Expected output file from COLONY with no errors where every spawn and egg fertilization occurred as observed and all information input to COLONY was sufficient.
(XLSX)

S12 Table COLONY output mode. Inferred male and female parent averaged over ten COLONY runs with different seed numbers.
(XLSX)

S13 Table COLONY output mode with error analysis. Error analysis for male and female parent assignment to offspring.
(XLSX)

S14 Table COLONY input for reduced return. Input file submitted to COLONY with reduced offspring in order to mimic the reduced returns observed in the wild.
(XLSX)

S15 Table Reduced return COLONY output mode with error analysis. Error analysis for male and female parent assignment to reduced offspring.
(XLSX)

S16 Table Error summary. Summary of all errors for all sample input and reduced offspring input.
(XLSX)

S17 Table Cost analysis. Costs for each reagent at each step of molecular work for Rapture analysis with 500 baits.
(XLSX)

## References

[1] National Marine Fisheries Service. Fisheries Economies of the United States 2012. U.S. Departmernt of Commerice, NOAA; 2014. https://ST/economics/publications/feus/fisheries_economics_2012

[2] Vanstone JW. An Annotated Ethnohistorical Bibliography of the Nushagak River Region, Alaska. Fieldiana Anthropol. 1968;54: 149–189.

[3] Boxberger DL. The Lummi Indians and the Canadian/American Pacific Salmon Treaty. Am Indian Q. 1988;12: 299–311. 10.2307/1184403

[4] Tsosie R. Land, Culture, and Community: Reflections on Native Sovereignty and Property in America Property, Wealth, & Inequality. Indiana Law Rev. 2000;34: 1291–1312.

[5] Hanna JM. “Oncorhynchus” spp.: Climate Change, Pacific Northwest Tribes, and Salmon. Nat Resour Environ. 2007;22: 13–17.

[6] Gunther E. An Analysis of the First Salmon Ceremony. Am Anthropol. 1926;28: 605–617.

[7] Swezey SL, Heizer RF. Ritual Management of Salmonid Fish Resources in California. 1977; 30.

[8] Cederholm CJ, Kunze MD, Murota T, Sibatani A. Pacific Salmon Carcasses: Essential Contributions of Nutrients and Energy for Aquatic and Terrestrial Ecosystems. Fisheries. 1999;24: 6–15.

[9] Rex JF, Petticrew EL. Delivery of marine-derived nutrients to streambeds by Pacific salmon. Nat Geosci. 2008;1: 840–843. 10.1038/ngeo364

[10] Quinn TP. The Behavior and Ecology of Pacific Salmon and Trout. University of Washington Press; 2018.

[11] Begg GA, Friedland KD, Pearce JB. Stock identification and its role in stock assessment and fisheries management: an overview. Fish Res. 1999;43: 1–8. 10.1016/S0165-7836(99)00062-4

[12] Paris JR, Sherman KD, Bell E, Boulenger C, Delord C, El-Mahdi MBM, et al Understanding and managing fish populations: keeping the toolbox fit for purpose. J Fish Biol. 2018;92: 727–751. 10.1111/jfb.13549 29537089

[13] Lichatowich J, Mobrand L, Lestelle L. Depletion and extinction of Pacific salmon (Oncorhynchus spp.): A different perspective. ICES J Mar Sci. 1999;56: 467–472. 10.1006/jmsc.1999.0457

[14] Gresh T, Lichatowich J, Schoonmaker P. An Estimation of Historic and Current Levels of Salmon Production in the Northeast Pacific Ecosystem: Evidence of a Nutrient Deficit in the Freshwater Systems of the Pacific Northwest. Fisheries. 2000;25: 15–21.

[15] Gustafson RG, Waples RS, Myers JM, Weitkamp LA, Bryant GJ, Johnson OW, et al Pacific Salmon Extinctions: Quantifying Lost and Remaining Diversity. Conserv Biol. 2007;21: 1009–1020. 10.1111/j.1523-1739.2007.00693.x 17650251

[16] Levin PS, Schiewe MH. Preserving Salmon Biodiversity: The number of Pacific salmon has declined dramatically. But the loss of genetic diversity may be a bigger problem. Am Sci. 2001;89: 220–227.

[17] Foley JA, DeFries R, Asner GP, Barford C, Bonan G, Carpenter SR, et al Global Consequences of Land Use. Science. 2005;309: 570–574. 10.1126/science.1111772 16040698

[18] NOAA Fisheries. Recovery Plan for The Evolutionarily Significant Units of Sacramento River Winter-run Chinook Salmon and Central Valley Spring-run Chinook Salmon and the DPS of California Central Valley Steelhead | NOAA Fisheries. 6 Apr 2018 [cited 1 May 2018]. www.fisheries.noaa.gov

[19] Fisheries N. Atlantic Herring Management Plan | NOAA Fisheries. 23 Oct 2018 [cited 3 Nov 2018]. /management-plan/atlantic-herring-management-plan

[20] North Pacific Anadromous Fish Commission. NPAFC Pacific salmonid hatchery release statistics. 2017 [cited 8 May 2018]. www.npafc.org

[21] Wolf KS, O’Neal JS. Tagging, Telemetry, and Marking Measures for Monitoring Fish Populations. Pacific Northwest Aquatic Monitoring Partnership Special Publication; 2010. http://www.rmpc.org/files/TTM_Compendium_2010.pdf

[22] Nandor GF, Longwill JR, Webb DL. Overview of the Coded Wire Tag Program. 2009; 53.

[23] Bergman PK, Haw F, Blankenship HL, Buckley RM. Perspectives on Design, Use, and Misuse of Fish Tags. Fisheries. 1992;17: 20–25.

[24] Gibbons WJ, Andrews KM. PIT Tagging: Simple Technology at Its Best. BioScience. 2004;54: 447–454.

[25] Chittenden CM, Butterworth KG, Cubitt KF, Jacobs MC, Ladouceur A, Welch DW, et al Maximum tag to body size ratios for an endangered coho salmon (O. kisutch) stock based on physiology and performance. Environ Biol Fishes. 2009;84: 129–140. 10.1007/s10641-008-9396-9

[26] Shaklee JB, Beacham TD, Seeb L, White BA. Managing fisheries using genetic data: case studies from four species of Pacific salmon. Fish Res. 1999;43: 45–78. 10.1016/S0165-7836(99)00066-1

[27] Campbell MR, Kozfkay CC, Copeland T, Schrader WC, Ackerman MW, Narum SR. Estimating Abundance and Life History Characteristics of Threatened Wild Snake River Steelhead Stocks by Using Genetic Stock Identification. Trans Am Fish Soc. 2012;141: 1310–1327. 10.1080/00028487.2012.690816

[28] Whitlock S l., Schultz L d., Schreck C b., Hess J e. Using genetic pedigree reconstruction to estimate effective spawner abundance from redd surveys: an example involving Pacific lamprey (Entosphenus tridentatus). Can J Fish Aquat Sci. 2017;74: 1646–1653. 10.1139/cjfas-2016-0154

[29] Beacham TD, Wallace C, MacConnachie C, Jonsen K, McIntosh B, Candy JR, et al Population and individual identification of coho salmon in British Columbia through parentage-based tagging and genetic stock identification: an alternative to coded-wire tags. Can J Fish Aquat Sci. 2017;74: 1391–1410. 10.1139/cjfas-2016-0452

[30] Anderson EC, Garza JC. The Power of Single-Nucleotide Polymorphisms for Large-Scale Parentage Inference. Genetics. 2006;172: 2567–2582. 10.1534/genetics.105.048074 16387880 PMC1456362

[31] Denson MR, B K IV, Jenkins WE, Darden TL. Assessing Red Drum Juvenile Stocking in a South Carolina Estuary Using Genetic Identification. North Am J Fish Manag. 2012;32: 32–43. 10.1080/02755947.2011.649577

[32] Ashton NK, Campbell MR, Anders PJ, Powell MS, Cain KD. Evaluating Microsatellite Markers for Parentage-based Tagging of Hatchery Burbot. Northwest Sci. 2016;90: 249–259.

[33] Webster MS, Reichart L. Use of microsatellites for parentage and kinship analyses in animals In: Zimmer EA, Roalson EH, editors. Molecular Evolution: Producing the Biochemical Data, Part B. Webster, Michael S.; Washington State Univ, Sch Biol Sci, Pullman, WA 99164 USA; 2005 pp. 222–238. 10.1016/S0076-6879(05)95014-3 15865970

[34] Steele CA, Anderson EC, Ackerman MW, Hess MA, Campbell NR, Narum SR, et al A validation of parentage-based tagging using hatchery steelhead in the Snake River basin. Can J Fish Aquat Sci. 2013;70: 1046–1054. 10.1139/cjfas-2012-0451

[35] Abadía-Cardoso A, Anderson EC, Pearse DE, Carlos Garza J. Large-scale parentage analysis reveals reproductive patterns and heritability of spawn timing in a hatchery population of steelhead (Oncorhynchus mykiss). Mol Ecol. 2013;22: 4733–4746. 10.1111/mec.12426 23962061

[36] Gill P. An assessment of the utility of single nucleotide polymorphisms (SNPs) for forensic purposes. Int J Legal Med. 2001;114: 204–210. 10.1007/s004149900117 11355396

[37] Baird NA, Etter PD, Atwood TS, Currey MC, Shiver AL, Lewis ZA, et al Rapid SNP Discovery and Genetic Mapping Using Sequenced RAD Markers. PLOS ONE. 2008;3: e3376 10.1371/journal.pone.0003376 18852878 PMC2557064

[38] Johnson GE, Khan F, Skalski JR, Klatte BA. Sluiceway Operations to Pass Juvenile Salmonids at The Dalles Dam, Columbia River, USA. North Am J Fish Manag. 2013;33: 1000–1012. 10.1080/02755947.2013.822441

[39] Ozerov M, Vasemägi A, Wennevik V, Diaz-Fernandez R, Kent M, Gilbey J, et al Finding Markers That Make a Difference: DNA Pooling and SNP-Arrays Identify Population Informative Markers for Genetic Stock Identification. PLoS One San Franc. 2013;8: e82434 10.1371/journal.pone.0082434 24358184 PMC3864958

[40] Pavey SA. High-throughput SNPs for all: genotyping-in-thousands. Mol Ecol Resour. 2015;15: 685–687. 10.1111/1755-0998.12405 26095005

[41] Campbell NR, Harmon SA, Narum SR. Genotyping-in-Thousands by sequencing (GT-seq): A cost effective SNP genotyping method based on custom amplicon sequencing. Mol Ecol Resour. 2015;15: 855–867. 10.1111/1755-0998.12357 25476721

[42] Ali OA, O’Rourke SM, Amish SJ, Meek MH, Luikart G, Jeffres C, et al RAD Capture (Rapture): Flexible and Efficient Sequence-Based Genotyping. Genetics. 2016;202: 389–400. 10.1534/genetics.115.183665 26715661 PMC4788223

[43] Glaubitz JC, Rhodes OE, Dewoody JA. Prospects for inferring pairwise relationships with single nucleotide polymorphisms. Mol Ecol. 2003;12: 1039–1047. 10.1046/j.1365-294x.2003.01790.x 12753222

[44] Hauser L, Baird M, Hilborn R, Seeb LW, Seeb JE. An empirical comparison of SNPs and microsatellites for parentage and kinship assignment in a wild sockeye salmon (Oncorhynchus nerka) population. Mol Ecol Resour. 2011;11: 150–161. 10.1111/j.1755-0998.2010.02961.x 21429171

[45] Crête-Lafrenière A, Weir LK, Bernatchez L. Framing the Salmonidae Family Phylogenetic Portrait: A More Complete Picture from Increased Taxon Sampling. PLoS One San Franc. 2012;7: e46662 10.1371/journal.pone.0046662 23071608 PMC3465342

[46] Prince DJ, O’Rourke SM, Thompson TQ, Ali OA, Lyman HS, Saglam IK, et al The evolutionary basis of premature migration in Pacific salmon highlights the utility of genomics for informing conservation. Sci Adv. 2017;3: e1603198 10.1126/sciadv.1603198 28835916 PMC5559211

[47] Korneliussen TS, Albrechtsen A, Nielsen R. ANGSD: Analysis of Next Generation Sequencing Data. BMC Bioinformatics. 2014;15: 356 10.1186/s12859-014-0356-4 25420514 PMC4248462

[48] Jones OR, Wang J. COLONY: a program for parentage and sibship inference from multilocus genotype data. Mol Ecol Resour. 2010;10: 551–555. 10.1111/j.1755-0998.2009.02787.x 21565056

[49] Hammer SA, Blankenship HL. Cost Comparison of Marks, Tags, and Mark-with-Tag Combinations Used in Salmonid Research. North Am J Aquac. 2001;63: 171–178.

[50] Kelson SJ, Miller MR, Thompson TQ, O’Rourke SM, Carlson SM. Do genomics and sex predict migration in a partially migratory salmonid fish, Oncorhynchus mykiss? Can J Fish Aquat Sci. 2019;76: 2080–2088. 10.1139/cjfas-2018-0394

[51] Kelson SJ, Miller MR, Thompson TQ, O’Rourke SM, Carlson SM. Temporal dynamics of migration-linked genetic variation are driven by streamflows and riverscape permeability. Mol Ecol. 2020;29: 870–885. 10.1111/mec.15367 32012393 PMC7078995

[52] Margres MJ, Jones ME, Epstein B, Kerlin DH, Comte S, Fox S, et al Large-effect loci affect survival in Tasmanian devils (Sarcophilus harrisii) infected with a transmissible cancer. Mol Ecol. 2018;27: 4189–4199. 10.1111/mec.14853 30171778 PMC6759049

[53] Araki H, Cooper B, Blouin MS. Genetic Effects of Captive Breeding Cause a Rapid, Cumulative Fitness Decline in the Wild. Science. 2007;318: 100–103. 10.1126/science.1145621 17916734

[54] Christie MR, Marine ML, French RA, Blouin MS. Genetic adaptation to captivity can occur in a single generation. Proc Natl Acad Sci U S A. 2012;109: 238–242. 10.1073/pnas.1111073109 22184236 PMC3252900

[55] Sommer TR, Harrell WC, Nobriga ML, Kurth R. Floodplain as Habitat for Native Fish: Lessons from California’s Yolo Bypass. 01; 7.

[56] Katz JVE, Jeffres C, Conrad L, Sommer T, Nick C, Martinez J, et al. Knaggs Ranch Experimental Agricultural Floodplain at Knaggs Ranch on Yolo Bypass. US Bureau of Reclamation; 2013. https://watershed.ucdavis.edu

[57] State of California. Fisheries Branch Research Permitting. 2018 [cited 6 May 2019]. https://www.wildlife.ca.gov/Explore/Organization/FB/Permitting#476861438-endangered-species-act-esa-4d-authorizations

[58] Miller MR, Brunelli JP, Wheeler PA, Liu S, Rexroad CE, Palti Y, et al A conserved haplotype controls parallel adaptation in geographically distant salmonid populations. Mol Ecol. 2012;21: 237–249. 10.1111/j.1365-294X.2011.05305.x 21988725 PMC3664428

[59] Stearley RF, Smith GR. Phylogeny of the Pacific Trouts and Salmons (Oncorhynchus) and Genera of the Family Salmonidae. Trans Am Fish Soc. 1993;122: 1–33.

[60] Berthelot C, Brunet F, Chalopin D, Juanchich A, Bernard M, Noël B, et al The rainbow trout genome provides novel insights into evolution after whole-genome duplication in vertebrates. Nat Commun. 2014;5: 3657 10.1038/ncomms4657 24755649 PMC4071752

[61] SAMtools. [cited 26 Apr 2018]. http://samtools.sourceforge.net/

[62] Buerkle CA, Gompert Z. Population genomics based on low coverage sequencing: how low should we go? Mol Ecol. 2013;22: 3028–3035. 10.1111/mec.12105 23174005

